# Social Facilitation of Laughter and Smiles in Preschool Children

**DOI:** 10.3389/fpsyg.2018.01048

**Published:** 2018-06-27

**Authors:** Caspar Addyman, Charlotte Fogelquist, Lenka Levakova, Sarah Rees

**Affiliations:** ^1^Department of Psychology, Goldsmiths, University of London, London, United Kingdom; ^2^Department of Psychological Sciences, Birkbeck, University of London, London, United Kingdom

**Keywords:** preschoolers, laughter, smiles, humor, peer groups

## Abstract

Surprisingly little is known about the social dimensions of laughter in preschool children. We studied children’s responses to amusing video clips in the presence or absence of peers. The sample consisted of 9 boys and 11 girls aged 31–49 months (*M* 39.8, *SD* 4.2) who watched three cartoons under three different conditions: individually, in pairs, or in groups of 6 or 8. The social viewing conditions showed significantly higher numbers of laughs and smiles than the individual viewing condition. On average children laughed eight times as much in company as on their own and smiled almost three times as much. No differences were found between pairs and groups, and no association was found between subjective funniness ratings and group size. This suggests that the presence of even a single social partner can change behavior in response to humorous material. It supports the idea that laughter and smiles are primarily flexible social signals rather than reflexive responses to humor.

## Introduction

Laughter is a universal aspect of human life occurring in almost all individuals and across all cultures (Provine, 2001; Martin, 2010). Laughter is a distinctive pattern of vocalization that is instantly recognizable and emerges by 4 months (Sroufe and Waters, 1976). Despite variations in cultural norms and across generations, the actual sounds of laughter are difficult to tell apart from one culture to the next (Gervais and Wilson, 2005). Laughter is also a highly social phenomenon (Chapman, 1973; Provine, 1993; Addyman and Addyman, 2013). Surprisingly few experiments have been conducted on the social dimensions of laughter in young children and how this relates to their responses to humor. In the current study, we sought to do so by adapting the methods of Chapman (1973) to measure smiling and laughter in preschoolers watching humorous videos on their own or in the company of their peers.

Noting that not all laughs are alike, Giles and Oxford (1970) proposed seven mutually exclusive categories of laughter. The most common types were humorous laughter, described as a behavioral response to amusing stimuli, and social laughter, described as a behavioral response allowing integration within a given social group. Social laughter occurs either as a direct response to other group members laughing or as a result of group expectations of laughter and, as such, serves to reduce social and cognitive discord, thereby promoting acceptance and loyalty within the group. Chapman and Wright (1976) point out that laughter, as distinct from smiling, in response to amusing stimuli, is relatively uncommon in the absence of another person to share the humor. Humor is not an easy thing to define or classify. It is difficult to pinpoint exactly what makes something funny (McGhee, 1979). Sometimes humor is defined in terms of ability to provoke laughter and sometimes the terms laughter and humor are used interchangeably (Devereux and Ginsburg, 2001). Studies have shown young children’s laughter to be correlated with subjective ratings of funniness (Chapman, 1983). However, people often smile and laugh in the absence of humor, and people may feel amused without smiling or laughing, particularly when alone (Weisfeld, 1993).

Darwin and others thought smiling and laughter were manifestations of degrees of intense happiness (Darwin, 1872; Ekman and Friesen, 1984). However, several studies support the contrasting hypothesis that smiling is primarily a social indication of friendliness. Kraut and Johnston (1979) observed people in a bowling alley. They found smiles were more likely when interacting with others than when scoring a strike. Fernández-Dols and Ruiz-Belda (1995) observed 22 gold medal winners at the presentation ceremony of the Barcelona Olympic Games. Medalists smiled most during face-to-face encounters associated with the actual presentation of their medals, but only sporadically during other parts of the presentation ceremony.

Comparative and phylogenetic studies support the notion that laughter and smiling are evolved instincts with a social purpose. Many primate species display a relaxed open-mouth “play face” during social play, frequently accompanied by a pant-like vocalization, both of which bear resemblance to human laughter (Provine, 2001; Caron, 2002). Many primate species also display a silent bared-teeth expression analogous to the human smile which, although believed to have originally been a sign of aggression, has evolved to function as a sign of non-hostility, appeasement and friendliness (Caron, 2002). Laughter even seems to be a signal for social play in rats that can be evoked by tickling (Knutson et al., 1998). More recently, Davila-Ross et al. (2009) tickled infants from all five great apes species and found that acoustic similarities in their laughter matched the known genetic similarities of the species. In other work, Davila-Ross et al. (2011) found that chimpanzees changed the form of their laughs and laughed more in social than solitary play situations. This supported similar field observations by Matsusaka (2004).

In humans, laughter and smiling are instinctive and spontaneous behaviors that begin at a very young age (Provine, 1996; Caron, 2002). Most smiles in 1–5-month-old infants happen in response to the human face or voice, suggesting it is primarily a social behavior (Sroufe and Waters, 1976). After crying, laughter is one of the earliest social vocalizations produced by human babies, and babies start to laugh in response to other people’s actions at around the age of 4 months (Martin, 2010). Most laughter in babies and young children is elicited through tactile stimulation as well as incongruous sights and sounds, so long as such incongruities are experienced in a secure or playful setting (Rothbart, 1973). Congenitally deaf and blind children emit appropriate laughter in social situations despite never having perceived laughter in others, suggesting this laughter is innate (Provine, 2001).

Infant smiles and laughter may communicate wellbeing. In Gartstein and Rothbart’s (2003) Infant Behavioral Questionnaire-Revised (IBQ-R) questions about smiles and laughter are combined into a single subscale that contributes to an overall score for positive affect. However the story is complex; Mireault et al. (2012) found that direct measures of smiling and laughter at 6 months did not correlate with this IBQ-R score. But the IBQ-R measure did predict greater attachment at a year, suggesting that ‘less good-humored infants elicit greater parental engagement’ (Mireault et al., 2012 p. 797). This suggests it is important to distinguish trait measures of “good humor” from state measures of laughter and smiling in response to humorous material or social cues.

Evidence for the sociality of laughter and smiling comes from a study by Provine and Fischer (1989) in which students were asked to keep laughter diaries in which they recorded all instances of laughing, smiling and talking in a given week. Results revealed that laughter was over 30 times, and smiling over six times, more likely to occur in social than in solitary situations. Provine (2001) proposes that laughter, rather like mutual grooming in primate troops, serves a non-linguistic function in creating social bonds, reinforcing friendships, and drawing people into the fold. During conversation, laughter seems to be synchronized into the speech stream in an orderly manner, a phenomenon known as the *punctuation effect*. Through covertly observing human interaction in a variety of everyday settings such as shopping malls, restaurants or bars, Provine (1993) recorded the amount of laughter in natural interactions. Rather than the expected results of the audience laughing more than the speaker, the opposite was true; laughter amongst the speakers being on average 46% higher than that of the audience. It was further noted that most of the speaker’s pre-laughter comments were not in the least humorous, leading Provine (2004) to suggest that the essential ingredient for laughter, rather than being a joke, is the presence of another person. Interestingly, Dezecache and Dunbar (2012) found that subgroups of shared laughter remained small (around 3–4 people) even as social groups became much larger.

A number of studies have linked humorous laughter to social group size. Morrison (1940) found a high positive correlation between audience size and the number of laughs elicited during a theater performance. Young and Frye (1966) found that undergraduates laughed more in response to a joke in groups than when alone, but humor ratings did not differ. Fridlund (1991) had participants watch an enjoyable video in four conditions of varying sociality: alone; alone but believing a friend close by was otherwise engaged; alone but believing a friend close by was watching the same videotape in a separate room; and with a friend present at the viewing. Smiling, assessed by electromyography activity of the underlying muscles of the cheek, was found to increase as a function of the degree of sociality of the viewing process, but was not associated with subjective ratings of emotion felt, leading to the conclusion that smiling is less dependent on emotion than on social context. In a similar study, Devereux and Ginsburg (2001) found laughter was more frequent and lasted longer when participants watched videos in pairs than when watching alone. No differences in subjective ratings of amusement or happiness felt, or funniness of video clip, were found, supporting the notion that laugher is a function of the sociality of a situation regardless of internal emotional state.

Classic observational studies of preschool children find laughter to be primarily social (Kenderdine, 1931; Brackett, 1933). Sherman (1975) coded videos of 596 formal lessons in a preschool. He found that glee, defined as joyful screaming, laughing and intense physical acts was highly contagious, spreading in a chain reaction. Jones and Raag (1989) observed infant play sessions and found that infants were not inclined to smile until turning around to make eye contact with their mothers. To investigate the extent to which laughter and smiling are socially facilitated, Chapman (1973) had 7–8 year-olds listen to humorous material through headphones under three conditions: alone; with a non-listening companion; and with a companion listening to the same material. Results revealed that total time engaged in overt laughing and smiling was higher in children accompanied by a listening companion than in those accompanied by a non-listening companion, and higher in children accompanied by a non-listening companion than in those listening alone. Children who laughed and smiled the most also gave the highest subjective ratings of funniness. A subsequent study, also with 7–8 year-olds, included a social exclusion condition (Chapman, 1975). Participants listened to humorous material with two confederates. Results revealed that the more the confederates made eye contact with each other, and therefore not with the participant, the less the participant laughed or smiled. This effect occurred independently of whether the participants believed they were listening to the same humorous material as the confederates. This supports the idea that it is the sharing of a social situation *per se*, rather than the sharing of humorous stimuli, that is the crucial factor in eliciting laughter and smiling in children.

A subsequent literature review revealed very little experimental research that had investigated the social facilitative aspects of laughter in preschool children. One aim of the present study was to investigate Chapman’s (1973) findings that children’s laughter increases in company with a much younger sample. A second aim was to see if group size changes this social effect. In the present study preschool children watched humorous videos alone or in pairs or in groups of six or eight. We predicted that incidents of smiling and laughter would increase in the social condition, with the effect being greater in the larger group. We predicted that the amount of laughter and smiling would not be related to children’s subjective ratings of funniness. Finally, in a social context it is useful to distinguish a non-vocal cue like smiling from laughter (Haakana, 2010). Therefore we treat smiling and laughter as separate variables.

## Materials and Methods

### Participants

Participants were 20 children (11 female) who attended a private preschool in Twickenham. Participants’ ages ranged from 2 years 7 months to 4 years 1 month (mean 39.8 months, SD 4.2 months). All children were British born and included 19 who were white and 1 who was mixed raced. All parents provided written consent to the children taking part and verbal consent was also obtained from the children prior to testing sessions. Ethical approval was obtained from the ethics committee at Birkbeck, University of London.

### Materials

Video clips from the Bernard Bear cartoon series were used as humorous material. The Bernard Bear series was chosen as it contains no dialog but relies on slapstick and incongruous humor which previous research has shown particularly appeals to children of preschool age (Rothbart, 1973). Each video consisted of two episodes and had a total running time of between 6 min 35 s and 7 min 43 s (see online materials for episode list). Video clips were presented using a Lenovo ThinkPad 2.0 laptop connected to a 56 cm Samsung Syncmaster flat television screen positioned on a table at a height of approximately 55 cm and at a distance from participants of approximately 1.5 meters. Participants were recorded via a built-in camera on the laptop as well as via a compact HD JVC camcorder placed on a tripod positioned just behind and to the right of the television screen. A Blue Snowball microphone was connected to the laptop and positioned on a shelf to the right of participants.

Subjective funniness ratings were taken using a printed visual scale containing simple cartoon-like pictures of a happy face meaning “very funny,” a neutral face meaning “quite funny” and a sad face meaning “not funny” (see online materials, Addyman et al., 2017).

### Design

The experiment used a 3 × 3 mixed design. The experimental independent variable was group size as a within subjects factor, a second between subjects independent variable counterbalanced viewing order. Children were randomly assigned to three viewing orders A, B, and C, and watched three videos individually, in pairs, or in groups of 6 or 8 on separate occasions (see **Table 1** and online materials). The two main dependent variables were the number of laughs and smiles elicited by the video clip in each child in each viewing condition. An additional dependent variable was the children’s subjective funniness ratings.

**Table 1:** Viewings of the funny video took place in three experimental conditions (individual, pairs or groups) that took place in three separate sessions with viewing order counterbalanced as shown.

VIEWING ORDER	GROUP SIZE	Session 1 Week 1–2 Video 1	Session 2 Week 3–4 Video 2	Session 3 Week 5–6 Video 3
A	6 (3m, 3f)	Individually	Pairs	Group
B	8 (4m, 4f)	Pairs	Group	Individually
C	6 (2m, 4f)	Group	Individually	Pairs

### Procedure

The study took place over several sessions over a 6-week period supervised by two researchers, one of whom worked at the preschool and was well known to all the children. Video and recording equipment were set up in an area of the preschool separated from the main area by 1.2-meter-high privacy screens. In the individual viewing condition, a researcher invited one child to come to watch a short video clip and made the child comfortable on cushions on the floor at approximately 1.5 meters from the television screen. Throughout the viewing of the video clip, both researchers were positioned just outside of the privacy screen, slightly behind and to the left of the child. This allowed the researchers to supervise and provide any necessary reassurance to the child, whilst remaining separate from the viewing process. Care was taken by the researchers throughout to maintain a neutral expression and not to be perceived as participating in the watching of the video clip. This procedure was then repeated for the next child until all children had been tested. In the pairs viewing condition, the same procedure as above was followed, except that children were seated side by side on floor cushions. In the group viewing condition, again, the same procedure was followed, except that children were seated in a semi-circle on floor cushions.

In all viewing conditions, once the video clip had ended, the researcher who worked at the preschool asked each child how funny they thought the video clip was, using the visual scale described above. Regardless of viewing condition, children were always asked individually. Finally, the child was invited to choose a sticker as a reward for taking part.

### Video Coding

Smiles and laughter were coded offline from the video recordings of the children. Video presentation software (Camtasia Studio 8) was used to allow the researchers to watch the recordings of participants simultaneously with the video clip being viewed. Laughs and smiles were operationalised based on the definitions of Chapman (1975). A laugh was defined as an audible inarticulate vocal sound and/or visible shaking of the shoulders or torso, whilst a smile was defined as an upward stretching of the corners of the mouth unaccompanied by vocal sound.

The three researchers each independently coded two thirds of videos across all viewing conditions, ensuring each video was coded twice. A 10-s timer was set to start 20 s after commencement of the video clip and to end once 6 min had elapsed. In each 10-s interval, the researchers noted the number of laughs and number of smiles per child on a coding sheet (see online materials). Once coding had been completed, the researchers compared their respective totals. The pairwise correlations of total smiles and total laughs per child per view condition between coders were all greater than 0.95. In cases where there were minor discrepancies in totals, the mean number of laughs and mean number of smiles were calculated and recorded on a master table of data. A minor discrepancy was a scoring difference between coders of 3 or fewer laughs or smiles per child per video. In a small number of cases where discrepancies were larger, the video clip was re-watched and a consensus reached. To further minimize bias or error, a colleague who was naïve to the study analyzed 15% of the recordings in the manner described above. A percentage of similarity between researcher coding and naïve coding was calculated by dividing the number of agreements between the researchers and naïve coder by the number of agreements plus number of disagreements between the researchers and naïve coder. The similarity percentage was found to be 86%.

## Results

To investigate the social role of laughter and smiles in preschool children watching funny videos, laughter, smiles and funniness ratings were looked at separately. All analysis was performed using the R statistics language, version 3.4.2 with ANOVA performed using CRAN packages ez, version 4.4.0 (Lawrence, 2016) and power calculations using package pwr, version 1.2.1 (Champely, 2017). The data, the analysis scripts and the code to generate all figures are provided in the online materials (Addyman et al., 2017). **Table 2** shows the descriptive statistics and **Table 3** the pairwise correlations for all the main experimental variables.

**Table 2:** Descriptive Statistics.

*Variable*	*N*	*Mean*	*SD*	*SE*	*Median*	*Min*	*Max*	*Range*	*Skewness*	*Kurtosis*
Age months	20	39.8	4.21	0.94	40	31	49	18	−0.2	−0.12
Laughs groups	20	8.2	8.14	1.82	6.25	0	27	27	0.75	−0.53
Laughs pairs	20	7.6	6.68	1.49	7.25	0	21	21	0.49	−0.96
Laughs indiv	20	0.92	2.36	0.53	0	0	10.5	10.5	3.33	10.62
Smiles groups	20	11.85	7.61	1.7	10.5	2	27.5	25.5	0.6	−0.9
Smiles pairs	20	11.38	7.45	1.66	9.75	2	28	26	0.71	−0.26
Smiles indiv	20	4.1	4.61	1.03	2.75	0.5	18	17.5	1.76	2.1

**Table 3:** Pairwise Pearson correlations between child age and laughter and smile totals per condition.

	Laughs groups	Laughs pairs	Laughs indiv	Smiles groups	Smiles pairs	Smiles indiv
Age months	0.163 (0.492)	−0.010 (0.968)	−0.206 (0.385)	0.088 (0.713)	0.300 (0.198)	0.063 (0.791)
Laughs groups		0.431 (0.058)	0.268 (0.253)	**0.586^∗∗^ (0.007)**	**0.615^∗^ (0.004)**	0.007 (0.977)
Laughs pairs			0.347 (0.133)	0.361 (0.118)	**0.529^∗^ (0.016)**	−0.108 (0.651)
Laughs indiv				0.145 (0.541)	−0.098 (0.681)	0.021 (0.929)
Smiles groups					**0.733^∗∗∗^ (<0.001)**	0.353 (0.127)
Smiles pairs						0.140 (0.556)

^∗^*p < 0.05,*^∗∗^*p < 0.01,*^∗∗∗^*p < 0.001. p-values are shown in brackets with significant correlations shown in bold.*

### Power

The F-scores reported in Chapman (1973) show large effect sizes of the group size variable for both laughter (${\text{η}}_{\text{p}}^{2}$ = 0.43) and smiling (${\text{η}}_{\text{p}}^{2}$ = 0.57). Using the smaller value we calculate that the necessary sample size to detect a similar effect with alpha level of 0.05 and power of 0.80 with our 3 × 3 repeated measures design would be 17. Our actual sample size of 20 participants gives a predicted power of 0.90 (see online materials for full calculations).

### Laughter

Descriptive statistics showed a greater number of laughs in the group viewing condition (*M* 8.20, *SD* 8.14) and in the pair viewing condition (*M* 7.60, *SD* 6.68) than in the individual viewing condition (*M* 0.93, *SD* 2.36). To test the experimental hypothesis, a mixed 3 × 3 ANOVA was conducted with group size as the within-subjects variable (group, pairs, individual) and viewing order as the between-subjects variable (orders A, B or C). Mauchly’s test of Sphericity was passed with *W* = 0.996, *p* = 0.761, therefore homogeneity of variance could be assumed.

**FIGURE 1:**
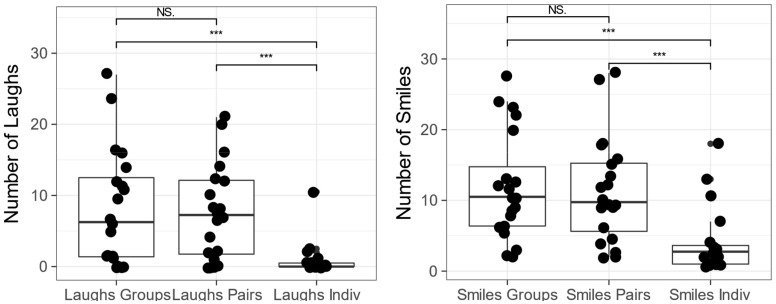
Tukey box plots of the number of laughs **(left)** and smiles **(right)** by condition. Each dot represents one child in one condition and the superimposed box plots show the median and inter-quartile range. Horizontal bars above the plot indicate significance levels of the paired-sample *t*-test planned comparison.

Results showed a highly significant main effect of viewing condition for laughs, *F*(2,34) = 12.93, *p* < 0.001 with a generalized eta squared, *ges* = 0.25. There was no main effect of viewing order *F*(2,17) = 0.44, *p* = 0.65, ges = 0.03 and no interaction *F*(4,34) = 1.85, *p* = 0.14, *ges* = 0.09. The difference in laughter between viewing conditions was compared with a set of Bonferroni corrected two-tailed, pairwise *t*-tests. These showed that children laughed significantly more in pairs than alone *t*(19) = 4.77, *p* < 0.001, and in groups than alone *t*(19) = 4.15, *p* < 0.001. However, the amount of laughter per child did not differ between pairs and groups *t*(19) = 0.33, *p* < 0.74. These results support the hypothesis that the amount of laughter is determined by the presence of a social partner and are shown in the left-hand panel of **Figure 1**.

### Smiles

Descriptive statistics showed a greater number of smiles in the group viewing condition (*M* 11.85, *SD* 7.61) and in the pair viewing condition (*M* 11.38, *SD* 7.45) than in the individual viewing condition (*M* 4.10, SD 4.61). The same 3 × 3 ANOVA as above was carried out. Mauchly’s test of Sphericity was passed with *W* = 0.703, *p* = 0.06, therefore homogeneity of variance could be assumed.

Results showed a highly significant main effect of viewing condition for smiles, *F*(2,34) = 16.31, *p* < 0.001, *ges* = 0.26. There was no main effect of viewing order *F*(2,17) = 1.43, *p* = 0.26, *ges* = 0.10, and no interaction *F*(4,34) = 1.90, *p* = 0.13, *ges* = 0.07. As before, group viewing conditions were compared with pair-wise *t*-tests. These showed that children smiled significantly more in pairs than alone *t*(19) = 3.92, *p* < 0.001, and in groups than when alone *t*(19) = 4.70, *p* < 0.001. However, the amount of laughter per child did not differ between pairs and groups *t*(19) = 0.39, *p* < 0.70. Again, these results support the hypothesis that the amount of smiling is determined by the presence of a social partner and are shown in the right-hand panel of **Figure 1**.

### Subjective Funniness Ratings

To investigate the association between children’s subjective funniness ratings and viewing condition, totals of “not funny,” “quite funny” and “very funny” ratings were calculated for each viewing condition. A Pearson’s Chi-Square test of association showed that there were no significant differences in subjective funniness ratings between the group, pair and individual viewing conditions, χ^2^ = 2.033, *d.f.* = 4, *p* = 0.73. Despite laughing and smiling more when watching in pairs and groups, children did not rate the videos in these conditions as more funny. A similar analysis revealed that all videos were considered equally funny, χ^2^ = 2.27, *d.f.* = 4, *p* = 0.69. These ratings are summarized in **Table 4**.

**Table 4:** Children’s funniness ratings for each video according to size of group watching (left) and based on content (right).

Funniness rating	Group size		
	Group	Pair	Indiv
Not funny	4	2	3
Quite funny	3	6	6
Very funny	13	12	11
Total	20	20	20

**Funniness rating**	**Video**		
	**1**	**2**	**3**

Not funny	3	3	3
Quite funny	3	7	5
Very funny	14	10	12
Total	20	20	20

**FIGURE 2:**
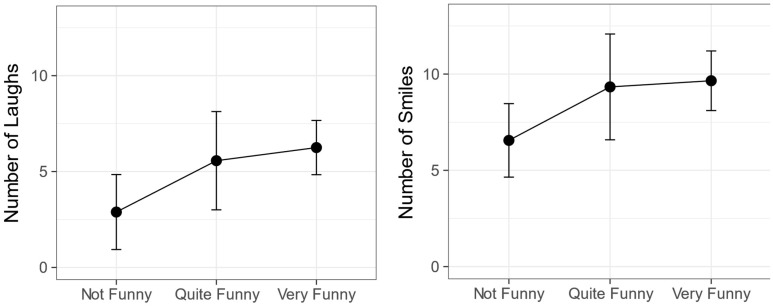
How laughs **(left)** and smiles **(right)** relate to subjective funniness. Error bars represent ±1 standard error.

Next, it was investigated whether subjective funniness ratings would predict the number of laughs and smiles. For each video the data were grouped according to whether each child had said the video was Not Funny, Quite Funny or Very Funny. The mean numbers of laughs and smiles for each of these groups were then calculated. A one-way ANOVA showed no relationship between number of laughs and funniness, *F*(2,31) = 0.21, *p* = 0.81, *ges* = 0.01. A similar one-way ANOVA showed no relationship between number of smiles and subjective funniness *F*(2,31) = 0.48, *p* = 0.63, *ges* = 0.03. The data are shown in **Figure 2**.

However, both these analyses may be overly conservative given that the funniness rating scale is ordinal and ratings are repeated measures across the three experimental conditions. Therefore, a further analysis was conducted using the repolr CRAN package, version 3.4 which fits logistic regression model to repeated ordinal scores, using a generalized estimating equation methodology (Parsons, 2016). The rating was the response variable and Group Size and Presentation Order were entered as ordinal predictor variables. This confirmed the findings of the previous analysis as there was no Group Size effect (model coefficient = −0.417, *p* = 0.52), no Order effect (coefficient = −1.220, *p* = 0.09) and no interaction (coefficient = 0.302, *p* = 0.41). Code for all these analyses is provided in the supporting materials.

## Discussion

This experiment investigated the influence of social peers on preschoolers’ responses to humorous materials. In line with predictions, we found that the presence of a social partner significantly increased smiling and laughter. When watching a funny cartoon, on average children laughed eight times more in company than when on their own, while smiles increased by a factor of around 2.8. The amount of laughter or smiling did not differ between pair or group conditions. This suggests that the presence of a single social partner can be sufficient to increase overt laughter and smiles. When children’s funniness ratings were taken into account, it was found that the greater amount of laughter and smiles in groups and pairs was not associated with higher ratings of funniness. Finally, there was no association between individual funniness ratings and the amount of laughter and smiles produced.

Chapman (1973) found that 7–8 year-olds laughed and smiled more in pairs than individually. The findings of the current study extend that result by looking at a much younger age group (mean age 3 years 4 months) and by including a group condition. Our experimental approach goes beyond the observational work on social laughter in adults (Kraut and Johnston, 1979; Fernández-Dols and Ruiz-Belda, 1995) and links to the related work on social laughter with adults (Fridlund, 1991; Young and Frye, 1966; Devereux and Ginsburg, 2001). The results of the present study provide a demonstration of the clear social role of overt laughter and smiles from a much younger age than shown in previous research. Furthermore, age was not correlated with any of the experimental measures suggesting this effect is already well established at this age. A challenge for future research would be to extend this method to younger ages.

The lack of difference in the amount of laughing and smiling between the pair and group conditions was unexpected. Mehu and Dunbar (2008) carried out naturalistic observations in public areas of people interacting in small groups in which group size, composition, in terms of sex and age of individuals, and social context of interactions were taken into account. Their results revealed group size to have the largest overall effect on the amount of laughter and smiling, with rates increasing as a function of group size. Group size had no influence in the current experiment, and the lack of difference between the pair and group conditions goes against a pure social contagion explanation. If children’s laughter and smiles increased in response to the smiles and laughs of others, higher scores would be expected in the group condition. This is at odds with the contagious properties of laughter in preschool children reported by Brackett (1933) and Sherman (1975), as well as with the experimental research using laugh boxes by Provine (1992) which found that laughter itself elicited laughter. One difference between this study and previous work is the relatively passive and non-social nature of the task. Children were watching a video rather than interacting with each other.

Non-statistical observations of our video data indicated that in the pair viewing condition, the laughter of one child did sometimes set the other off laughing, and that in the group viewing condition, this happened in a kind of chain reaction. Incongruous events (for example, Bernard Bear getting stuck in a bin) elicited high levels of laughter but rather than all children bursting into laughter simultaneously, it was often the initial laughter of one particularly gregarious child that quickly spread. Likewise, observations indicated that children in pairs or groups frequently made eye contact with each other whilst laughing. In the individual condition, smiling children would sometimes try to catch the eye of the researchers, presumably to share the joke. These non-statistical observations correspond with previous research. Jones and Raag (1989) found that infants engaged in play tend only to smile when turning to make eye contact with carers while Kraut and Johnston (1979) and Fernández-Dols and Ruiz-Belda (1995) found that most adult smiling occurs during face-to-face contact.

Should laughter and smiling be treated as a single construct? This is both a theoretical and methodological question. When assessing temperament it is reasonable to combine them as indicators for positive affect as in the IBQ-R (Gartstein and Rothbart, 2003). In social and communicative setting it is worth keeping the distinction (Haakana, 2010) but this requires clear operational definitions and measures. In the current experiment, it was noted that often a smile would become a laugh, and often a laugh would end with a smile and most inconsistencies between coders concerned laughs being termed smiles and vice versa. With our 10 s blocked counting, consensus was high between our coders and our results do show a stronger effect in the laughter compared to the smiles. But our method does not allow us to account for different intensities of laughter in terms of volume or duration, or for different intensities of smiling. Likewise, analyses of eye contact, laughter initiation and contagion were not possible in the current study which relied on a single microphone and single camera angle in a noisy environment. Future studies should use multiple cameras and individual lapel microphones to record data for richer time-series analyses. Future work should also include temperament measures.

In Chapman’s (1973) study, children who laughed and smiled the most also gave the highest subjective ratings of funniness. This was not found in the current study. The videos were rated Very Funny by most children in all viewing conditions and the Chi-Squared tests and logistic regression found no association between funniness of videos and viewing conditions. Likewise, despite the apparent trends seen in **Figure 2**, statistical analysis revealed that there were no more laughter and smiles in cases rated funnier by children. One explanation may be that these very young children did not fully understand what they were being asked. McGhee (1977) suggests that use of five- or seven-point funniness rating scales, whilst appropriate for older children, may not be appropriate for younger children. In the current study, a three-point funniness rating scale was used. Whilst it was the view of the researcher who knew the children that most could easily do this task, children’s responses were often quite arbitrary, therefore calling into question the validity of relying on subjective ratings in children so young. Another possibility is that the study was underpowered to detect these effects. Future work should include a control task with non-funny stimuli to ensure children can answer this question.

Chapman (1975) emphasized that the unassuming nature of young children make them ideal participants for investigating spontaneous behaviors such as laughter. One of the main strengths of the current experiment was its high ecological validity. It was conducted during children’s normal day-to-day preschool activities and took place in a screened-off corner of the main room of the preschool. One downside was inevitably some background noise from other activities, but it is not believed the children were unduly affected by this. The upside was that the children remained in a familiar setting and so no children felt anxious, and this also helped keep them naïve to the fact they were being observed or evaluated, thereby maintaining ecological validity. Throughout the experiment, the researchers stood just outside of the privacy screens and slightly behind the children, which meant that children in the individual viewing condition were not alone in the strictest sense. This was required due to the children’s young age and preschool regulations that require adult supervision at all times. The fact that laughter was minimal in the “individual” condition suggests the children acted as if watching alone or that children respond differently in the presence of an adult than a co-viewing peer. Finally, all the children in this study were well known to each other, having attended the same preschool for an extended period, increasing any likely social effects. Future research could investigate if the current effect is modulated by friendship and peer relations as is found for prosocial behaviors (Sebanc, 2003).

Giles and Oxford (1970) proposed that social laughter and humorous laughter are mutually exclusive. The findings of the current experiment suggest that laughter and smiling have a strongly social role even in a humorous setting. An earlier study of preschoolers measured laughter in response to a humorous recording either alone or after observing a laughing or non-laughing peer (Brown et al., 1980). That study found a mixed pattern of results in that laughing increased across conditions but smiling occurred least after encountering a non-smiling peer. This led those authors to favor an imitation learning account of their results (Bandura, 1978). If that were the case in our experiment we would expect far more laughter and smiling in the large group condition where there are more peers to copy. Instead our results favor an account in terms of social facilitation (Zajonc, 1965) where presence of even a single social partner greatly increases laughter and smiles.

Extensive work by Elena Hoicka and colleagues has investigated humor production and understanding in preschool children. They have shown that infants and preschoolers can understand and produce humor (Hoicka and Gattis, 2008; Hoicka and Akhtar, 2012). They have also shown that preschoolers can tell jokes from pretending, and apply contextual cues to understand humor (Hoicka and Akhtar, 2011; Hoicka and Butcher, 2016). However, the primarily social aspect of laughter and smiles found in the present study does not diminish preschoolers’ appreciation and understanding of humor (Hoicka and Akhtar, 2012; Hoicka and Butcher, 2016) The children in the present study found the cartoons funny in all viewing conditions but their laughter and smiles were strongly modulated by social setting. Our study shows that indexing young children’s understanding of humor with laughter is not straightforward, but the cognitive skills children require for understanding humor make it a fascinating lens onto preschool development.

Certainly, more research is needed to understand how social and emotional factors interact with learning in preschoolers. Many researchers now recognize that emotion is an indivisible part of the preschool experience. Social and Emotional Learning (SEL) has become a well-known acronym with early years literature (Morris et al., 2013; Williams et al., 2014). Whilst that literature addresses aggression and warmth, it rarely directly considers laughter, mirth or glee. Rana Esseily and colleagues recently demonstrated that laughter aided observational learning in 18-month-old infants (Esseily et al., 2016). This suggests there is potential to recruit young children’s natural mirth and glee and the social setting of the preschool to enhance learning. Humor in social settings could have a pedagogical role in preschool. For example, it would be interesting to investigate whether the observational learning benefit of laughter found by Esseily et al. (2016) translated to greater comprehension and learning from videos in a setting like that of the current experiment.

The current study adds to the body of recent work that suggests primarily social function for both smiles and laughter, particularly in development. Ramsey and Gentzler (2015) propose that expressions of positive affect and positive close relationships have a bidirectional and reciprocal relationship during development, leading to the expectation that shared laughter strengthens friendships and friendships strengthen laughter. While a recent twin study of infants age 6–12 months goes further and finds a strong environmental influence of parental positive affect on infant laughter and smiling (Planalp et al., 2017). Finally, recent mathematical modeling work of adult smiles and laughter suggest they have multiple social functions conveying reward, affiliation and dominance (Martin et al., 2017; Wood et al., 2017). Future developmental research should look for these differences in young children.

## Conclusion

In conclusion, the present study has demonstrated that social presence of peers makes a large difference to preschoolers’ overt laughter and smiling, but that increased ostensive signals of humor appreciation are not related to the perceived funniness of humorous material. Given the importance of social laughter and smiling in establishing social bonds and the value of humor within the context of cognitive development, it is hoped that the current experiment will form the basis for further investigation into the social nature of laughter and smiling in preschool children.

## Author Contributions

CF, LL, and SR ran the study and coded the video data. CA ran the analysis. CA and SR wrote the final report with input from CF and LL. All authors were responsible for the design and planning of the study.

## Conflict of Interest Statement

The authors declare that the research was conducted in the absence of any commercial or financial relationships that could be construed as a potential conflict of interest.
